# Supplementary material for: Physiological Variability during Prehospital Stroke Care: Which Monitoring and Interventions Are Used?
Source: Healthcare (Basel). 2024 Apr 15;12(8):835. doi: 10.3390/healthcare12080835 (PMC11050416; doi:10.3390/healthcare12080835)
Supplement: Supplementary file 1 [file healthcare-12-00835-s001.zip › Supplementary material table S1.pdf]

Supplementary material table S1

Table S1: Search strategy

<table>
<tr><td>Ovid MEDLINE(R) ALL <1946 to February 07, 2022></td><td>EBSCO CINAHL Tuesday, February 08, 2022 3:33:09 PM</td></tr>
<tr><td>1 (prehospital$ or pre-hospital$ or pre hospital$ or ambulance$ or paramedic$ or EMS).tw. 106247

2 Emergency Medical Services/ 148969

3 PHEM.mp. 90

4 Emergency Responders/ 9522

5 "out of hospital".mp. 36924

6 or/1-5 252394

7 CEREBROVASCULAR DISORDERS/ or exp BASAL GANGLIA CEREBROVASCULAR DISEASE/ or exp BRAIN ISCHEMIA/ or exp CAROTID ARTERY DISEASES/ or exp</td><td>
<table>
<tr><th>#</th><th>Query</th><th>Results</th></tr>
<tr><td>S1</td><td>prehospital* or pre-hospital* or pre hospital* or ambulance* or paramedic* or EMS</td><td>Display</td></tr>
<tr><td>S2</td><td>(MH "Emergency Medical Services")</td><td>Display</td></tr>
<tr><td>S3</td><td>PHEM</td><td>Display</td></tr>
<tr><td>S4</td><td>""out of hospital""</td><td>Display</td></tr>
<tr><td>S5</td><td>(MH "Emergency Medical Technicians")</td><td>Display</td></tr>
<tr><td>S6</td><td>(MH "Prehospital Care")</td><td>Display</td></tr>
<tr><td>S7</td><td>S1 OR S2 OR S3 OR S4 OR S5 OR S6</td><td>Display</td></tr>
<tr><td>S8</td><td>MH CEREBROVASCULAR DISORDERS OR MH BASAL GANGLIA CEREBROVASCULAR DISEASE OR MH BRAIN ISCHEMIA OR MH CAROTID ARTERY DISEASES OR MH NTRACRANIAL ARTERIAL DISEASES OR MH ("INTRACRANIAL EMBOLISM AND THROMBOSIS") OR MH INTRACRANIAL HEMORRHAGES</td><td>Display</td></tr>
<tr><td>S9</td><td>(((stroke OR poststroke OR post-stroke OR cerebrovasc* OR brain) AND (vasc* OR cerebral)) AND (vasc* OR cva* OR apoplex* OR SAH))</td><td>Display</td></tr>
<tr><td>S10</td><td>((brain* OR cerebr* OR cerebell* OR intracran* OR intracerebral) N5 (isch?emi*</td><td>Display</td></tr>
</table>
</td></tr>
</table>

INTRACRANIAL ARTERIAL DISEASES/ or exp INTRACRANIAL ARTERIOVENOUS MALFORMATIONS/ or exp "INTRACRANIAL EMBOLISM AND THROMBOSIS"/ or exp INTRACRANIAL HEMORRHAGES/ or STROKE/ or exp BRAIN INFARCTION/ or STROKE, LACUNAR/ or VASOSPASM, INTRACRANIAL/ or VERTEBRAL ARTERY DISSECTION/ or BRAIN INJURIES/ or BRAIN INJURY, CHRONIC/ 1585800

8 (stroke or poststroke or post-stroke or cerebrovasc* or brain vasc* or cerebral vasc* or cva* or apoplex* or SAH).ti,ab. 871396

9 ((brain* or cerebr* or cerebell* or intracran* or intracerebral) adj5 (isch?emi* or infarct* or thrombo* or emboli* or occlus*)).ti,ab. 282389

10 ((brain* or cerebr* or cerebell* or intracerebral or intracranial or subarachnoid) adj5 (haemorrhage* or hemorrhage* or haematoma* or

	OR infarct* OR thrombo* OR emboli* OR occlus*))	
S11	((brain* OR cerebr* OR cerebell* OR intracerebral OR intracranial OR subarachnoid) N5 (haemorrhage* OR hemorrhage* OR haematoma* OR hematoma* OR bleed*))	Display
S12	S8 OR S9 OR S10 OR S11	Display
S13	(MH "Hemodynamics") OR "h?emodynamic*"	Display
S14	(MH "Blood Pressure") OR "blood pressure"	Display
S15	(MH "Heart Rate") OR "heart rate"	Display
S16	(MH "Carbon Dioxide") OR "carbon dioxide"	Display
S17	(MH "Vital Signs") OR "vital sign*"	Display
S18	(MH "Cerebrovascular Circulation")	Display
S19	cerebral n2 autoregulat*	Display
S20	(MH "Capnography") OR "capnograph*"	Display
S21	(MH "Hypocapnia") OR "hypocapni*"	Display
S22	(MH "Hypercapnia") OR "hypercapni*"	Display
S23	((diastolic or systolic or pulse) n2 pressure)	Display
S24	(MH "Hypertension") OR "hypertension"	Display
S25	(MH "Hypotension") OR "Hypotension"	Display
S26	cardiac rate	Display
S27	(MH "Tachycardia") OR "tachycardia"	Display
S28	(MH "Bradycardia") OR "bradycardia"	Display
S29	(MH "Oxygenation") OR "Oxygenation"	Display
S30	""End tidal CO2""	Display
S31	"capnometr*"	Display
S32	(MH "Oximetry") OR "Oximetr*"	Display

hematoma* or bleed*)).ti,ab. 180998

11 7 or 8 or 9 or 10 2039098

12 h?emodynamic*.mp. or Hemodynamic Monitoring/ or Hemodynamics/ 608178

13 blood pressure.mp. or Blood Pressure/ 1125369

14 heart rate.mp. or Heart Rate/ 580820

15 carbon dioxide.mp. or Carbon Dioxide/ 288641

16 vital sign*.mp. or Vital Signs/ 58941

17 (cerebral adj2 autoregulat*).mp. or Cerebrovascular Circulation/ [mp=ti, ab, hw, tn, ot, dm, mf, dv, kf, fx, dq, nm, ox, px, rx, ui, sy] 78400

S33	(MH "Oxygen Saturation") OR "Oxygen Saturation"	Display
S34	S13 OR S14 OR S15 OR S16 OR S17 OR S18 OR S19 OR S20 OR S21 OR S22 OR S23 OR S24 OR S25 OR S26 OR S27 OR S28 OR S29 OR S30 OR S31 OR S32 OR S33	Display
S35	S7 AND S12 AND S34	Display
S36	S13 OR S18 OR S19	31,465
S37	S14 OR S15 OR S16 OR S17 OR S20 OR S21 OR S22 OR S23 OR S24 OR S25 OR S26 OR S27 OR S28 OR S29 OR S30 OR S31 OR S32 OR S33	294,009
S38	S7 AND S12 AND S36 AND S37	21

18 Capnography/ or capnograph*.mp. [mp=ti, ab, hw, tn, ot, dm, mf, dv, kf, fx, dq, nm, ox, px, rx, ui, sy] 9229<br><br>19 Hypocapnia/ or hypocapni*.mp. 8114<br><br>20 Hypercapnia/ or hypercapni*.mp. 42457<br><br>21 ((diastolic or systolic or pulse) adj2 pressure).mp. [mp=ti, ab, hw, tn, ot, dm, mf, dv, kf, fx, dq, nm, ox, px, rx, ui, sy] 385008<br><br>22 Hypertension/ or hypertension.mp. 1509563<br><br>23 hypotension.mp. or Hypotension/ 247186<br><br>24 cardiac rate.mp. 1554<br><br>25 tachycardia.mp. or exp Tachycardia/ 265759	

26 bradycardia.mp. or Bradycardia/ 94556	
27 Oxygenation.mp. 185368	
28 "End tidal CO2".mp. 7217	
29 capnometr*.mp. [mp=ti, ab, hw, tn, ot, dm, mf, dv, kf, fx, dq, nm, ox, px, rx, ui, sy] 4103	
30 Oximetry/ 24615	
31 Oximetr*.mp. 51416	
32 Oxygen Saturation.mp. 117013	
33 or/12-32 3789242	
34 6 and 11 and 33 4367	
35 12 or 17 673323	
36 or/13-16,18-32 3424650	
37 6 and 11 and 35 and 36 447	

38 (prehospital$ or pre-hospital$ or pre hospital$ or ambulance$ or paramedic$ or EMS).tw. 106247<br><br>39 emergency health service/ 154496<br><br>40 rescue personnel/ or ambulance/ 28863<br><br>41 PHEM.mp. 90<br><br>42 "out of hospital".mp. 36924<br><br>43 or/38-42 259271<br><br>44 exp "CEREBROVASCULAR ACCIDENT"/ 409597<br><br>45 (stroke or poststroke or post-stroke or cerebrovasc* or brain vasc* or cerebral vasc* or cva* or apoplex* or SAH).ti,ab. 871396<br><br>46 ((brain* or cerebr* or cerebell* or intracran* or intracerebral) adj5	

(isch?emi* or infarct* or thrombo* or emboli* or occlus*)).ti,ab. 282389<br><br>47 ((brain* or cerebr* or cerebell* or intracerebral or intracranial or subarachnoid) adj5 (haemorrhage* or hemorrhage* or haematoma* or hematoma* or bleed*)).ti,ab. 180998<br><br>48 CEREBROVASCULAR DISORDERS/ or exp BASAL GANGLIA CEREBROVASCULAR DISEASE/ or exp BRAIN ISCHEMIA/ or exp CAROTID ARTERY DISEASES/ or exp INTRACRANIAL ARTERIAL DISEASES/ or exp "INTRACRANIAL EMBOLISM AND THROMBOSIS"/ or exp INTRACRANIAL HEMORRHAGES/ or STROKE/ or exp BRAIN INFARCTION/ or exp VERTEBRAL ARTERY DISSECTION/ 1469442<br><br>49 or/44-48 1959782<br><br>50 hemodynamics/ or h?emodynamic*.mp. 608178	

51 hemodynamic monitoring/ 16709<br><br>52 blood pressure.mp. or blood pressure/ 1125369<br><br>53 heart rate.mp. or heart rate/ 580820<br><br>54 carbon dioxide.mp. or carbon dioxide/ 288641<br><br>55 vital sign/ or vital sign*.mp. 58941<br><br>56 brain blood flow/ or autoregulation/ 195646<br><br>57 (cerebral adj2 autoregulat*).mp. [mp=ti, ab, hw, tn, ot, dm, mf, dv, kf, fx, dq, nm, ox, px, rx, ui, sy] 7085<br><br>58 capnograph*.mp. or capnography/ 9229	

59 hypocapnia/ or hypocapni*.mp. 8114<br><br>60 hypercapnia/ or hypercapni*.mp. 42457<br><br>61 ((diastolic or systolic or pulse) adj2 pressure).mp. 385008<br><br>62 diastolic blood pressure/ 105556<br><br>63 systolic blood pressure/ 175833<br><br>64 hypertension.mp. or hypertension/ 1509563<br><br>65 hypotension/ or Hypotension.mp. 247186<br><br>66 cardiac rate.mp. 1554<br><br>67 tachycardia/ or tachycardia.mp. 260033	

68 bradycardia.mp. or bradycardia/ 94556<br><br>69 Oxygenation.mp. or blood oxygenation/ 185368<br><br>70 end tidal carbon dioxide tension/ 9249<br><br>71 "End tidal CO2".mp. 7217<br><br>72 capnometry/ or capnometr*.mp. 6493<br><br>73 oximetry/ or Oximetr*.mp. 51416<br><br>74 oxygen saturation.mp. or oxygen saturation/ 117013<br><br>75 pulse oximetry/ 31959<br><br>76 or/50-75 3880549<br><br>77 43 and 49 and 76 4189	

78 50 or 51 or 56 or 57 784516<br><br>79 or/52-55,58-75 3420610<br><br>80 43 and 49 and 78 and 79 445<br><br>81 37 or 80 509<br><br>82 remove duplicates from 81 448	
