# Supplementary material for: Physiological Variability during Prehospital Stroke Care: Which Monitoring and Interventions Are Used?
Source: Healthcare (Basel). 2024 Apr 15;12(8):835. doi: 10.3390/healthcare12080835 (PMC11050416; doi:10.3390/healthcare12080835)
Supplement: Supplementary file 1 [file healthcare-12-00835-s001.zip › Supplementary material Figure S1.pdf]

Supplementary material Figure S1

Figure S1: Newcastle–Ottawa scale scores

	Selection			Comparability		Outcome				
Study	Representativeness of exposed cohort	Selection of non-exposed cohort	Ascertainment of exposure	Demonstration that outcome of interest was not present at start of study	Study controls for the most important factor	Study controls for any additional factor	Assessment of outcome	Was follow-up long enough for outcomes to occur	Adequacy of follow up of cohorts	Total Score
Fan 2015	*	*	*	*	*	*	*	*	*	9
Hatcher 2017	*	*	*	*	*	*	*	*	*	9
Slavin 2018	*	*	*	*	*	*	*	*	*	9
Atsumi 2019	*	*	*	*	*	*	*	*	*	9
Tsou 2019	*	*	*	*	*	*	*	*	*	9
Larsen 2022	*	*	*	*	*	*	*	*	*	9
Rodriguez-Luna 2018	*	*	*	*	*	*	*	*	*	9
Gioia 2016	*	*	*	*	*	.	*	*	*	8
Kench 2020	N/A	N/A	N/A	N/A	N/A	N/A	N/A	N/A	N/A	N/A
Fouche 2020	*	*	*	*	*	*	*	*	*	9
Asaithambi 2012	*	*	*	*	.	.	*	*	*	7

Kobsa 2021	N/A	N/A	N/A	N/A	N/A	N/A	N/A	N/A	N/A	N/A
Yperzeele 2016	*	.	*	*	*	*	*	*	*	8
Tran 2017	N/A	N/A	N/A	N/A	N/A	N/A	N/A	N/A	N/A	N/A
Ankoelar 2013	*	*	*	*	*	.	*	*	*	8
Bath 2019 (RIGHT2	*	*	*	*	*	*	*	*	*	9
Shaw 2014	*	*	*	*	N/A	N/A	*	*	*	7
Saver 2015	*	*	*	*	*	*	*	*	*	9
Dylla 2020	*	*	*	*	*	*	*	*	*	9
